# Validation of tissue factor pathway inhibitor 2 as a specific biomarker for preoperative prediction of clear cell carcinoma of the ovary

**DOI:** 10.1007/s10147-021-01914-y

**Published:** 2021-05-19

**Authors:** Etsuko Miyagi, Noriaki Arakawa, Kentaro Sakamaki, Naho Ruiz Yokota, Takeharu Yamanaka, Yuki Yamada, Satoshi Yamaguchi, Shoji Nagao, Yasuyuki Hirashima, Yuka Kasamatsu, Hisamori Kato, Tae Mogami, Yohei Miyagi, Hiroshi Kobayashi

**Affiliations:** 1 Department of Obstetrics and Gynecology, Yokohama City University Graduate School of Medicine, 3-9 Fukuura, Kanazawa-ku, Yokohama, Kanagawa 236-0004 Japan; 2 Department of Medical Life Science, Graduate School of Medical Life Science, Yokohama City University, Yokohama, Japan; 3 Division of Medicinal Safety Science, National Institute of Health Sciences, Kawasaki, Japan; 4 Department of Biostatistics, Center for Novel and Explanatory Clinical Trials (Y-NEXT), Yokohama, Japan; 5 Center for Data Science, Yokohama City University, Yokohama, Japan; 6 Department of Obstetrics and Gynecology, Nara Medical University, Nara, Japan; 7 Department of Gynecology, Hyogo Cancer Center, Hyogo, Japan; 8 Department of Gynecology, Shizuoka Cancer Center, Shizuoka, Japan; 9 Department of Gynecology, Kanagawa Cancer Center Research Institute, Yokohama, Japan; 10 Molecular Pathology and Genetics Division, Kanagawa Cancer Center, Yokohama, Japan

**Keywords:** Ovarian cancer, Clear cell carcinoma, TFPI2, Serum tumor marker, CA125

## Abstract

**Background:**

Tissue factor pathway inhibitor 2 (TFPI2) is a novel serum biomarker that discriminates ovarian clear cell carcinoma (CCC) from borderline ovarian tumors (BOTs) and non-clear cell epithelial ovarian cancers (EOCs). Here, we examined the performance of TFPI2 for preoperative diagnosis of CCC.

**Methods:**

Serum samples were obtained preoperatively from patients with ovarian masses, who needed surgical treatment at five hospitals in Japan. The diagnostic powers of TFPI2 and cancer antigen 125 (CA125) serum levels to discriminate CCC from BOTs, other EOCs, and benign lesions were compared.

**Results:**

A total of 351 patients including 69 CCCs were analyzed. Serum TFPI2 levels were significantly higher in CCC patients (mean ± SD, 508.2 ± 812.0 pg/mL) than in patients with benign lesions (154.7 ± 46.5), BOTs (181 ± 95.5) and other EOCs (265.4 ± 289.1). TFPI2 had a high diagnostic specificity for CCC (79.5%). In patients with benign ovarian endometriosis, no patient was positive for TFPI2, but 71.4% (15/21) were CA125 positive. TFPI2 showed good performance in discriminating stage II–IV CCC from BOTs and other EOCs (AUC 0.815 for TFPI2 versus 0.505 for CA125) or endometriosis (AUC 0.957 for TFPI2 versus 0.748 for CA125). The diagnostic sensitivity of TFPI2 to discriminate CCC from BOTs and other EOCs was improved from 43.5 to 71.0% when combined with CA125.

**Conclusions:**

High specificity of TFPI2 for preoperative detection of CCC was verified with the defined cutoff level of TFPI2 in clinical practice. TFPI2 and CA125 may contribute substantially to precise prediction of intractable CCC.

**Supplementary Information:**

The online version contains supplementary material available at 10.1007/s10147-021-01914-y.

## Introduction

Ovarian cancer is currently one of the most lethal gynecological malignancies and the eighth most common cancer in women worldwide. The estimated number of new ovarian cancer patients worldwide in 2018 was nearly 300,000 and the age-standardized incidence rate per 100,000 was 6.6 [1]. In Japan, a reported 10,048 women were newly diagnosed with ovarian cancer and 4745 women died of ovarian cancer in 2017 [2]. Thus, the age-standardized incidence rate has gradually increased from 6.5 (1994) to 9.0 (2014) over 2 decades [2]. In accordance with the annual patients report for 2015 based on the database of the Japan Society of Obstetrics and Gynecology, a high frequency (above 20%) of clear cell carcinoma was detected among epithelial cancers in Japanese women [3] compared with 10% in accordance with the International Federation of Gynecology and Obstetrics Committee report [4].

Clear cell carcinoma (CCC) of the ovary is an endometriosis-associated epithelial ovarian epithelial cancer (EOC) that has specific characteristics compared with serous carcinoma. CCC often exhibits resistance against standard chemotherapies such as paclitaxel and carboplatin, which leads to lower survival rates of CCC patients compared with patients with chemo-sensitive ovarian serous carcinoma [4–7]. CCC patients often show low or normal levels of serum cancer CA125 that has the highest sensitivity to detect high grade serous carcinoma [8, 9]. Furthermore, patients with benign ovarian endometriosis frequently show high serum CA125 levels [10]. These characteristics make preoperative diagnosis of CCC difficult.

To identify specific serum biomarkers of CCC, we focused on a serine protease inhibitor, tissue factor pathway inhibitor 2 (TFPI2; also known as placental protein 5) [11], as a candidate specific serum biomarker. [12, 13] A modified proteomics technique, “secretome,” was used to identify TFPI2 in media conditioned by CCC-derived cell lines [13]. We recently developed a highly efficient automated enzyme-linked immunosorbent assay system for TFPI2 detection and determined the adequate cutoff level of serum TFPI2 to discriminate patients with CCC from other epithelial ovarian cancers and borderline tumors or benign ovarian lesions including endometriosis [13]. Here, we validated the actual performance of TFPI2 as a specific serum biomarker for preoperative prediction of CCC in a multicenter study.

## Patients and methods

This study involved five hospitals in which more than 20 patients with EOC were treated in 1 year. Preoperative patients diagnosed with adnexal masses, which had surgical treatment indication because of symptoms and/or the need for histological diagnoses, were enrolled in this study from July 2016 to April 2018. In two institutes (Yokohama City University Hospital and Nara Medical University Hospital), reserved preoperative serum samples obtained from EOC patients, from January 2014 to July 2016, were used in this study because the samples were adequately drawn preoperatively and stored for analysis of TFPI2 serum concentrations. All patients underwent at least surgical removal of adnexal masses, and histological diagnoses were made by pathologists at each hospital using the WHO classification of tumors of the ovary 2014 [14]. All patients diagnosed with BOT or EOC were staged (I–IV) in accordance with the International Federation of Gynecology and Obstetrics 2014 guidelines [5] after the first operation. This study was performed in accordance with the Declaration of Helsinki and the Ethical Guidelines for Medical and Health Research Involving Human Subjects after approval by the Institutional Ethics Committee of Yokohama City University (B160602003).

Blood samples were drawn within 1 month of surgery and collected in Venoject II serum separator tubes (VP-AS109K60, Terumo, Tokyo, Japan). The tubes were stored for 2–3 h at 4 °C or 30 min at room temperature and then centrifuged at 1000–1500*g* for 10 min. Serum aliquots were stored at −40 to 80 °C. All samples were transported from each institute to the Yokohama City University Bio-Bank in dry ice and stored at −80 °C. TFPI2 and CA125 concentrations in each serum sample were measured at the same time at the department of clinical laboratory in Yokohama City University Hospital using reagents provided by Tosoh diagnostics product divisions (Tosoh Corporation, Tokyo, Japan). Measurements were performed by clinical laboratory technologists who were blinded to the study. The TFPI2 concentration was measured by the direct assay method on an automated immunoassay analyzer system (Tosoh Corporation) as described in our previous study [13]. The cutoff level of TFPI2 was 270 pg/mL in accordance with our previous study. [13] CA125 was also measured by the automated immunoassay analyzer system and a diagnostic reagent (E test TOSOH II) with a cutoff level of 35 U/mL.

The sample size was determined in accordance with the Japanese guideline to apply for extracorporeal diagnostic medicines in Japan, which requires more than 150 samples including non-targeted samples and the participation of more than two facilities. As a result, we calculated the sample number and at least 50 CCC and 150 non-clear EOC or borderline ovarian tumor (BOT) samples were needed to achieve the primary endpoint of this study, which was 80% specificity to detect CCC among other EOCs and BOTs, considering the number of patients in Japan [2, 3].

The non-parametric Mann–Whitney *U* test was used to evaluate differences between groups and subgroups. Receiver operating characteristic curves were constructed for serum TFPI2 and CA125 by plotting sensitivity versus (100 specificity), and areas under the receiver operating characteristic curves were calculated when discriminating patients with CCC from patients with other ovarian disease groups, patients with BOT and patients with other subtype epithelial cancers, and patients with ovarian endometriosis. The cutoff values of TFPI2 were predefined using the Youden index based on data from our previous exploratory study [13]. Sensitivity, specificity, positive and negative predictive values, and accuracy were determined for the predefined cutoff values. Data were analyzed using SAS ver. 9.4 (SAS Institute, Inc., Cary, NC) and Microsoft Excel software.

## Results

Among the 351 eligible serum samples included in this study, 77 were benign ovarian lesions, 65 were BOTs, and 209 were EOCs, which included 69 CCC cases. Among the 77 benign lesions that needed surgery, 21 samples were obtained from patients with ovarian endometriosis (Table 1). In the CCC subtype, 69.6% (48 of 69) of patients were diagnosed as stage I and 30.4% were within stage II–IV. Conversely, among the serous subtypes, 54 were high grade and most patients (89.6%) were diagnosed as stage III–IV. Detailed data of the study participants are summarized in Supplementary Table 1.

**Table 1:** Demographics of patients with ovarian tumors (*n* = 351)

Characteristics			
Age, years (*n* = 351)			
Mean ± SD	56.2 ± 14.6		
Median (range)	56 (20–93)		
Tumor size, cm (*n* = 351)			
Mean ± SD	125.2 ± 67.6		
Median (range)	120 (8–400)		
Blood biochemistry			
Albumin, g/mL (*n* = 351)			
Mean ± SD	4.0 ± 0.6		
Median (range)	4.2 (1.6–5.2)		
Creatinine, mg/dl (*n* = 351)			
Mean ± SD	0.6 ± 0.2		
Median (range)	0.6 (0.3–2.2)		
Benign lesion (*n* = 77)	*n* (%)		
Endometriosis	21 (27.3)		
Non-endometriosis	56 (72.7)		
Borderline tumor (*n* = 65)		FIGO stage, *n* (%)	
Histologic type, *n* (%)		Stage I	Stage II–IV
Clear cell	0 (0)	0	0
Serous	17 (26.2)	13 (76.5)	4 (23.5)
Endometrioid	3 (4.6)	3 (100)	0
Mucinous	38 (58.5)	38 (100)	0
Others	7 (10.8)	7 (100)	0
Epithelial ovarian cancer (*n* = 209)		FIGO stage, *n* (%)	
Histologic type, *n* (%)		Stage I	Stage II–IV
Clear cell	69 (33.0)	48 (69.6)	21 (30.4)
Serous	67 (32.1)	7 (10.4)	60 (89.6)
Endometrioid	31 (14.8)	23 (74.2)	8 (25.8)
Mucinous	24 (11.5)	18 (75.0)	6 (25.0)
Others	18 (8.6)	5 (27.8)	13 (72.2)

*FIGO* the International Federation of Gynecology and Obstetrics

**Fig. 1:**
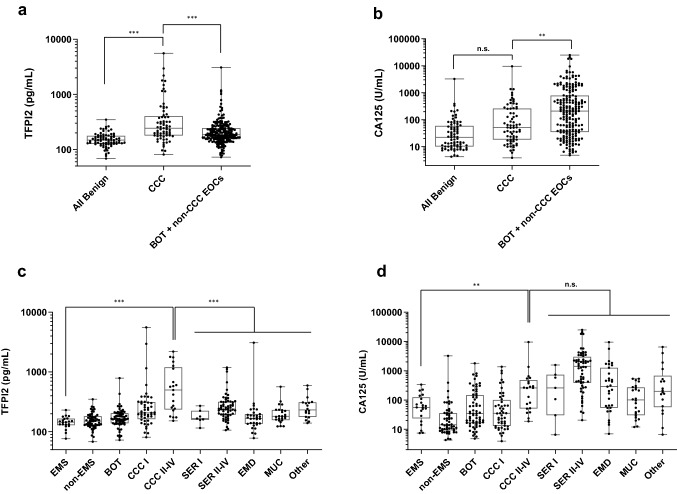
Distribution of serum tissue factor pathway inhibitor 2 (TFPI2) and CA125 in patients with ovarian lesions. Serum TFPI2 levels (**a**) and CA125 levels (**b**) in samples from patients with benign lesions (*n* = 77), borderline ovarian tumors (*n* = 65), clear cell carcinoma (*n* = 69), non-clear cell epithelial ovarian carcinoma (*n* = 140), and borderline ovarian tumors + non clear cell epithelial ovarian carcinoma (*n* = 205). Serum TFPI2 levels (**c**) and CA125 levels (**d**) in samples from patients with ovarian endometriosis (*n* = 21), non-endometriosis benign tumor (*n* = 56), stage I clear cell carcinoma (*n* = 48), stage II–IV clear cell carcinoma (*n* = 21), serous carcinoma stage I (*n* = 7), serous carcinoma stage II–IV (*n* = 60), endometrioid carcinoma (*n* = 31), mucinous carcinoma (*n* = 24), and other epithelial ovarian cancer (*n* = 18). Box shows 25th, 50th (median values), and 75th and whiskers indicate max and min values; ^**^p < 0.001, ^***^p < 0.0001 obtained by the Mann–Whitney *U* test. *n.s* not significant, *TFPI2* tissue factor pathway inhibitor 2, *CCC* clear cell carcinoma, *BOT* borderline ovarian tumor, *EOC* epithelial ovarian carcinoma, *EMS* ovarian endometriosis, *SER* serous carcinoma, *EMC* endometrioid carcinoma, *MUC* mucinous carcinoma

Values of serum TFPI2 and CA125 in each patient group are shown in Fig. 1 as box-and-whisker diagrams and described in Supplementary Table 2. Significantly higher levels of serum TFPI2 were found in CCC patients (mean ± SD: 508.2 ± 812.0 pg/mL) compared with patients with benign lesions (154.7 ± 46.5 pg/mL, *p* < 0.0001), endometriosis (145.5 ± 33.7 pg/mL, *p* < 0.0001), the non-clear cell EOC group (265.4 ± 289.1 pg/mL, *p* = 0.0124), and non-clear cell EOC + BOT group (238.6 ± 247.7 pg/mL, *p* = 0.0001) (Fig. 1a and Supplementary Table 2). Conversely, serum CA125 levels were significantly lower in all CCC patients (316.4 ± 167.0/mL) than non-clear cell EOC (1621.0 ± 3652.0 U/mL, *p* < 0.0001) and non-clear cell EOC + BOT (1152.0 ± 3096.0 U/mL) patients (*p* = 0.0001) (Fig. 1b and Supplementary Table 2). Furthermore, no significant difference in the CA125 level was found between all CCC and endometriosis patients (85.5 ± 84.8 U/mL, *p* = 0.782) (Fig. 1b and Supplementary Table 2).

The value of TFPI2 in patients with stage I CCC was relatively low (median 207 pg/mL). However, subgroup analyses in accordance with tumor stages revealed that TFPI2 levels were higher in stage II–IV CCC patients (median 499.9 pg/mL) than other groups (Fig. 1c and Supplementary Table 2). Among the 77 patients with benign ovarian lesions, only one patient with benign serous adenoma, who had a complication of chronic renal dysfunction (serum creatinine: 2.2 mg/dl), showed a positive result for TFPI2, but all patients with endometriosis (*n* = 21) were below the cutoff level (Fig. 1c and Supplementary Table 2). Conversely, CA125 levels were elevated in stage II–IV CCC (mean ± SD: 730.6 ± 2048.0 U/mL) as well as other epithelial cancer subgroups. Furthermore, a high CA125 level (> 35 U/mL) was often observed even in endometriosis and non-endometriosis benign patients (Fig. 1d and Supplementary Table 2).

**Fig. 2:**
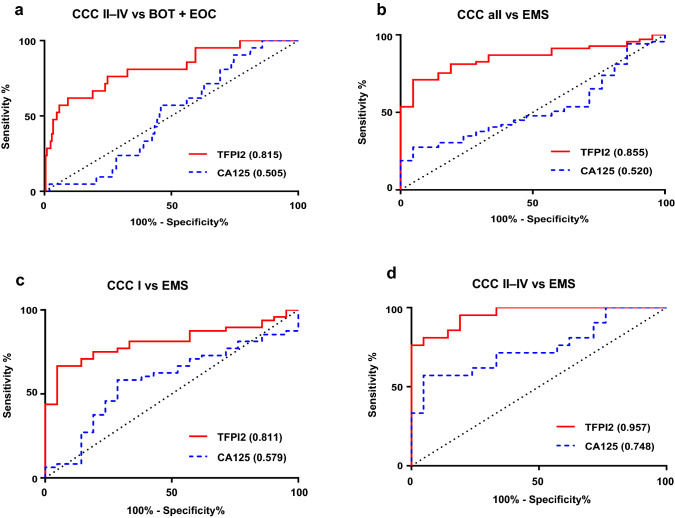
Receiver operating characteristic curves and areas under the curve (AUC) values of tissue factor pathway inhibitor 2 (TFPI2) and serum CA125 in discriminating clear cell carcinoma from other ovarian lesions. **a**; in patients with stage II–IV clear cell carcinoma vs. borderline ovarian tumor + epithelial ovarian carcinoma, **b**; clear cell carcinoma vs. ovarian endometriosis, **c**; clear cell carcinoma stage I vs endometriosis**, d**; clear cell carcinoma stage II–IV vs. endometriosis. *TFPI2* tissue factor pathway inhibitor 2, *CCC* clear cell carcinoma, *BOT* borderline ovarian tumor, *EOC* epithelial ovarian carcinoma, *EMS* endometriosis

To validate the diagnostic power of TFPI2, we compared area under the curve values obtained by receiver operating characteristic curves, sensitivities, specificities, positive and negative predictive values, and accuracies with the same indicators from our previous study [13] (Fig. 2 and Table 2), which unified the cutoff value as 270 pg/mL. To discriminate all CCC patients from BOT and other EOC patients, the specificity of TFPI2, which was evaluated as the primary endpoint of this study, was 79.5% (95% CI 73.3–84.8). This result showed that TFPI2 had a higher specificity compared with CA125 (24.9%, 95% CI 19.1–31.4), which was in good agreement with our previous study (85.1%). [13] In the current study, TFPI2 showed a high diagnostic power for stage II–IV CCC patients (AUC 0.815, 95% CI: 0.711–0.920) (Fig. 2a and Supplementary Table 3). Additionally, TFPI2 (AUC = 0.855, 95% CI 0.778–0.933) was superior to CA125 (AUC = 0.520, 95% CI 0.392–0.650) in discrimination of CCC patients, even at stage I (AUC of TFPI2: 0.811; AUC of CA125: 0.579), from endometriosis patients (Fig. 2b–d and Supplementary Table 4).

**Table 2:** Comparison of performances of tissue factor pathway inhibitor 2 (TFPI12) and CA125 in discriminating ovarian clear cell carcinoma patients from other patient groups

Validation	Discrimination (*N*)	Serum marker	AUC (95% CI)	Sensitivity % (95% CI)	Specificity % (95% CI)	PPV % (95% CI)	NPV % (95% CI)	Accuracy % (95% CI)
Current study	All CCC (69) vs BOT (65) + non-CCC EOC (140)	TFPI2	0.652 (0.572–0.732)	43.5 (31.6–56.0)	79.5 (73.3–84.8)	41.7 (30.2–53.9)	80.7 (74.6–85.9)	70.4 (64.7–75.8)
		CA125	0.654 (0.583–0.724)	62.3 (49.8–73.7)	24.9 (19.1–31.4)	21.8 (16.3–28.3)	66.2 (54.6–76.6)	34.3 (28.7–40.3)
Previous study [13]	All CCC (29) vs BOT (8) + non-CCC EOC (79)	TFPI2	0.854 (0.761–0.952)	79.3 (60.3–92.0)	85.1 (75.8–91.8)	63.9 (46.2–79.2)	92.5 (84.4–97.2)	83.6 (75.6–89.8)
		CA125	0.639 (0.533–0.745)	79.3 (60.3–92.0)	18.4 (10.9–28.1)	25.6 (16.2–34.4)	72.7 (49.8–89.3)	33.6 (25.1–43.0)
Current study	All CCC (69) vs EMS (21)	TFPI2	0.855 (0.778–0.933)	43.5 (31.6–56.0)	100 (83.9–100)	100 (88.4–100)	35.0 (23.1–48.4)	56.7 (45.8–67.1)
		CA125	0.520 (0.392–0.650)	62.3 (49.8–73.7)	28.6 (11.3–52.2)	74.1 (61.0–84.7)	18.8 (7.2–36.4)	54.4 (43.6–65.0)
Previous study [13]	All CCC (29) vs EMS (71)	TFPI2	0.924 (0.843–0.997)	82.8 (64.2–94.2)	93.0 (84.3–97.7)	82.8 (64.2–94.2)	93.0 (84.3–97.7)	90.0 (82.4–95.1)
		CA125	0.700 (0.584–0.817)	79.3 (60.3–92.0)	43.7 (31.9–56.0)	36.5 (24.7–49.6)	83.8 (68.0–93.8)	54.0 (43.7–64.0)

*TFPI2* tissue factor pathway inhibitor 2, *CCC* clear cell carcinoma, *BOT* borderline ovarian tumor, *EOC* epithelial ovarian carcinoma, *EMS* ovarian endometriosis, *PPV* positive predictive value, *NPV* negative predictive value, *AUC* area under the curve

Considering both high specificities of TFPI2 and high sensitivities of CA125 to predict CCC preoperatively in patients with ovarian lesions, we evaluated the indicators when positive was defined as TFPI2 or CA125 above cutoff levels and negative was defined as both TFPI2 and CA125 below cutoff levels (Table 3). The sensitivity of TFPI2 to detect CCC among BOT and EOC patients was 43.5% (95% CI 31.6–56.0), but the sensitivity was improved to 71.0% (95% CI 58.8–81.3) by combined analysis of TFPI2 and CA125 (Table 3) from 43.5% by TFPI2 alone (Table 2). The sensitivities of TFPI2 to detect stage I and II–IV CCC were 33.3% (95% CI 20.4–48.4) and 66.7% (95% CI 43.0–85.4), respectively (Table 2). However, they were improved to 60.4% (95% CI 45.3–74.2) and 95.2% (95% CI 76.2–99.9), respectively, by combined analysis of TFPI2 and CA125 (Table 3).

**Table 3:** Evaluation of performance of the combination of tissue factor pathway inhibitor 2 (TFPI12) and CA125 in discriminating clear cell carcinoma patients from other patient groups

Discrimination (no. of samples)	Serum marker (cutoff value)	Sensitivity % (95% CI)	Specificity % (95% CI)	PPV % (95% CI)	NPV % (95% CI)	Accuracy % (95% CI)
All CCC (69) vs BOT + non-CCC EOCs (205)	TFPI2 (270 pg/mL) or CA125 (35 U/mL)	71.0 (58.8–81.3)	23.9 (18.2–30.3)	23.9 (18.2–30.3)	71.0 (58.8–81.3)	35.8 (30.1–41.8)
CCC Stage I (48) or II–IV (21) vs BOT + non-CCC EOCs (205)	TFPI2 (270 pg/mL) or CA125 (35 U/mL)	Stage I: 60.4 (45.3–74.2)	Stage I: 23.9 (18.2–30.3)	Stage I: 15.7 (10.8–21.7)	Stage I: 72.1 (59.9–82.2)	Stage I: 30.8 (25.2–36.9)
		Stage II–IV: 95.2 (76.2–99.9)	Stage II–IV: 23.9 (18.2–30.3)	Stage II–IV: 11.4 (7.1–17.0)	Stage II–IV: 98.0 (89.4–100)	Stage II–IV: 30.5 (24.6–37.0)
CCC Stage I (48) or II–IV (21) vs EMS (21)	TFPI2 (270 pg/mL) or CA125 (35 U/mL)	Stage I: 60.4 (45.3–74.2)	Stage I: 28.6 (11.3–52.2)	Stage I: 65.9 (50.1–79.5)	Stage I: 24.0 (9.4–45.1)	Stage I: 50.7 (38.4–63.0)
		Stage II–IV: 95.2 (76.2–99.9)	Stage II–IV: 28.6 (11.3–52.2)	Stage II–IV: 57.1 (39.4–73.7)	Stage II–IV: 85.7 (42.1–99.6)	Stage II–IV: 61.9 (45.6–76.4)

*TFPI2* tissue factor pathway inhibitor 2, *CCC* clear cell carcinoma, *BOT* borderline ovarian tumor, *EOC* epithelial ovarian carcinoma, *EMS* ovarian endometriosis

## Discussion

In this study, we verified high specificity of TFPI2 to detect CCC and discriminate CCC from other ovarian lesions. The clinical needs of developing a new biomarker to detect and discriminate CCC from other ovarian tumors are based on two facts. First, chemotherapy shows limited efficacy in patients with advanced CCC because of its chemoresistant nature. [7, 15, 16] Therefore, optimal surgery is needed for CCC patients at the time of their primary treatment. Second, patients with ovarian endometriosis, which is the origin of CCC, often have high serum CA125 levels [17].

Endometriosis occurs in about 5–10% of women in their reproductive ages worldwide [17]. Japanese women are susceptible to endometriosis because of the characteristics of the social environment, such as low usage of oral contraceptives, late marriage, and a declining childbirth. Thus, the rate of CCC among EOCs has increased from 23.4 to 29.1% between 2002 and 2010 [18]. Our exploration to investigate the characteristics of ovarian CCC using cell lines identified TFPI2 by an original secretome-based method [13]. Next, we developed a precise TFPI2-measuring system and determined the cutoff level as 270 pg/mL through industry-academia joint research [13].

In clinical settings, CA125 is a non-specific, but sensitive serum tumor marker to detect EOC [10, 19, 20] and TFPI2 will be simultaneously evaluated to predict CCC preoperatively. Low sensitivity of TFPI2 in stage I CCC patients (33.3%) was found in this study. However, CA125 was positive in 50% of the same patient group. The combination of TFPI2 and CA125 was able to increase the sensitivity to detect stage I CCC up to 60%. In 1990, Jacob et al. reported a formula with sensitivity of 85% and specificity of 97% as a risk of malignancy index that incorporated CA125, ultrasound, and menopausal status [19]. However, the utility to predict CCC is unknown. TFPI2, which completely discriminated CCC from endometriosis in contrast to CA125, may work when both serum markers are evaluated together. We speculate that careful monitoring of both CA125 and TFPI2 may lead to early detection of transformation from endometriosis to ovarian CCC over time in patients with endometriosis. Furthermore, we consider that CA125, TFPI2, and imaging parameters obtained from ultrasound and MRI may be useful for more precise prediction of ovarian tumor types. However, in this study, the diagnoses of MRI imaging among CCC patients were “not ruled out malignancy” in 36 cases (52%) and “suggestive of malignancy” in 33 cases (47%). At present, we are developing an accurate prediction system for CCC, especially stage I patients, using a deep learning system of multimodalities by artificial intelligence.

Regarding the molecular characteristics of TFPI2, the CCC-specific elevation of serum TFPI2 was in accordance with its mRNA expression in both ovarian CCC cell lines and tumor tissues in our previous study [13]. Interestingly, previous studies have shown that methylation of cytosine–phosphorothioate–guanine islands located in the promoter region of TFPI2 gene reduces or abolishes expression of TFPI2 in cancers such as oral squamous cell carcinoma [21], diffuse B-cell lymphoma [22], hepatocellular, and pancreatic cancer. [23, 24] This silencing of TFPI2 through promoter cytosine–phosphorothioate–guanine methylation has been widely reported to be associated with elevated cancer cell invasion and progression in many types of cancers including breast cancers and gliomas [25]. Furthermore, promoter methylation of TFPI2 is a poor prognostic factor of hepatocellular carcinoma. [26] These results suggest why serum TFPI2 has not been identified or used as a candidate tumor marker for cancers other than ovarian CCC. In contrast to the tumor suppressor-like features of TFPI2, there is a report that demonstrates a paradoxical pro-invasive function of TFPI2 in hepatocellular carcinoma cells in vitro [27]. In this report, the pro-invasive effect of TFPI2 was associated with its binding to tissue factor-activated coagulation factor VII complex. We have reported that factor VII expression occurs frequently in EOC together with tissue factor, particularly in CCC cells [28]. TFPI2 was first identified as a canonical inhibitor of the tissue factor-activated coagulation factor VII complex that has serine protease activity and initiates the extrinsic blood coagulation cascade [29]. We also cloned the cDNA of TFPI2 and found markedly high expression of TFPI2 in the placenta [12, 30]. Recombinant TFPI2 inhibits the amidolytic activity of blood coagulation factors that include the tissue factor-activated coagulation factor VII complex, factor Xa, and factor XIa [31]. Thus, TFPI2 appears to function in the balance of blood coagulation–fibrinolysis systems under physiological conditions. We speculate that ectopic expression of TFPI2 in malignant tissues may perturb the balance. Notably, CCC patients have the highest risk of deep vein thrombosis caused by the hyper-coagulation status, known as “Trousseau syndrome” [32]. In this context, we have demonstrated secretion of extracellular vesicles rich in tissue factor–factor VII complex from ovarian cancer cells including CCC cells [33]. To clarify whether high serum TFPI2 levels in CCC patients are relevant to the thrombogenic condition, in vivo and in vitro research is needed.

This study has three major limitations. First, the data were generated from only preoperative serum samples obtained from patients with ovarian masses, who needed surgical treatment. Therefore, the dynamic changes accompanied by treatment are unknown. We are conducting a prospective follow-up study to clarify the changes in serum TFPI2 and CA125 levels after the surgery and during chemotherapy. The second limitation is that we have not identified the mechanism underlying the high level of serum TFPI2 in patients with ovarian tumors other than CCC. Four patients with advanced high grade serous carcinoma manifestation massive pleural and/or ascites showed very high levels of TFPI2 (1018–7649 pg/mL) together with high CA125 (380–16,587 U/mL). These patients were accompanied by massive ascites. It is possible that the origin of TFPI2 was not the cancer cells in these patients. To clarify this phenomenon, another study using immunohistochemistry is undergoing to investigate the origin of TFPI2 in patients with advanced high-grade serous carcinoma. The third limitation is that a central pathological diagnosis was not planned in the protocol because we only focused on the data in real clinical settings. However, comparison of the pathological diagnoses made by pathologists in each facility with the specialists and comparing the values of TFPI2 should be adopted in the next study.

In conclusion, we verified TFPI2 as a highly specific preoperative biomarker to predict CCC in clinical practice. The distinct characteristics of TFPI2 and CA125 may offer substantial contributions to adequate management of patients with intractable CCC. Additionally, we are going to apply TFPI2 to insurance coverage in Japan together with more practical clinical data.

## Supplementary Information

Below is the link to the electronic supplementary material.

Supplementary file1 (DOCX 26 KB)
