# Supplementary material for: Validation of tissue factor pathway inhibitor 2 as a specific biomarker for preoperative prediction of clear cell carcinoma of the ovary
Source: Int J Clin Oncol. 2021 May 19;26(7):1336–44. doi: 10.1007/s10147-021-01914-y (PMC8213588; doi:10.1007/s10147-021-01914-y)
Supplement: Supplementary file 1 — Supplementary file1 (DOCX 26 KB) [file 10147_2021_1914_MOESM1_ESM.docx]

| **Supplementary Table 1** Detailed characteristics of patients with ovarian tumors (n=351) | | | | | |
| --- | --- | --- | --- | --- | --- |
|  | **Number (%)** | **Age, years Median (range)** | **Tumor size, cm Median (range)** | **Albumin, g/ml Median (range)** | **Creatinine, mg/dl Median (range)** |
| Benign tumor (n=77) | 77 (100） | 51 (20–90) | 91 (35–295) | 4.3 (3.2–4.9) | 0.60 (0.43–4.6) |
| Endometriosis | 21 (27.3) | 43 (34–71) | 68 (35–160) | 4.4 (3.6–4.9) | 0.66 (0.47–0.78) |
| Non-EMS benign lesions | 56 (72.7) | 58 (20–90) | 121.5 (40–295) | 4.3 (3.2–4.8) | 0.60 (0.43–2.19) |
| Borderline ovarian tumors (n=65) | 65 (100） | 58 (23–87) | 150 (20–350) | 4.2 (2.9–5.2) | 0.62 (0.44–1.09) |
| Clear cell | 0 (0) | ― | ― | ― | ― |
| Serous | 17 (26.2) | 54 (26–76) | 100 (47–245) | 4.3 (3.6–4.7) | 0.62 (0.4–0.88) |
| Endometrioid | 3 (4.6) | 64 (53–82) | 80 (55–130) | 4.1 (3.9–4.6) | 0.64 (0.58–0.67) |
| Mucinous | 38 (58.5) | 57.5 (23–87) | 150 (20–350) | 4.2 (2.9–5.2) | 0.62 (0.44–1.09) |
| Other BOT | 7 (10.8) | 56 (32–80) | 60 (30–240) | 4.4 (3.8–4.7) | 0.66 (0.44–0.78) |
| Epithelial ovarian cancer (n=209) | 209 (100) | 57 (21–93) | 120 (8–400) | 4.0 (1.6–5.0) | 0.60 (0.29–1.80) |
| Stage I CCC | 48 (69.6) | 56.5 (35–73) | 135 (8–350) | 4.2 (2.3–5.0) | 0.62 (0.41–1.14) |
| Stage II–IV CCC | 21 (30.4) | 64 (43–81) | 140 (40–240) | 3.4 (2.1–4.8) | 0.57 (0.36–0.86) |
| Stage I Serous carcinoma | 7 (10.4) | 61 (48–93) | 120 (70–210) | 4.3 (3.3–4.6) | 0.54 (0.42–0.71) |
| Stage II–IV Serous carcinoma | 60 (89.6) | 66 (42–86) | 80 (10–250) | 3.85 (1.6–4.7) | 0.60 (0.29–1.80) |
| Endometrial carcinomas | 31 (14.8) | 53 (32–87) | 120 (20–300) | 4.2 (2.7–4.8) | 0.53 (0.38–0.94) |
| Mucinous carcinomas | 24 (11.5) | 50 (21–79) | 200 (100–400) | 4.05 (2.0–4.8) | 0.585 (0.33–1.54) |
| Other EOC | 18 (8.6) | 55 (36–83) | 110 (50–214) | 3.85 (2.7–4.4) | 0.605 (0.42–1.04) |
| EMS, endometriosis; CCC, clear cell carcinoma; TFPI2, tissue factor pathway inhibitor 2; CA125, serum cancer antigen 125; BOT, borderline ovarian tumor; EOC, epithelial ovarian carcinoma. | | | | | |

| **Supplementary Table 2** Serum levels of tissue factor pathway inhibitor 2 and CA125 in patients with benign ovarian lesions, borderline tumors, clear cell carcinomas, and non-clear cell epithelial ovarian cancers | | | | | |
| --- | --- | --- | --- | --- | --- |
|  | **TFPI2 (pg/mL)** | |  | **CA125 (U/mL)** | |
|  | **Median (range)** | **Mean±SD** |  | **Median (range)** | **Mean±SD** |
| Groups |  |  |  |  |  |
| Benign lesion | 147.8 (68.3-348.8) | 154.7±46.5 |  | 22.4 (4.3-3246.0) | 90.6±371.1 |
| Borderline tumor (BOT) | 164.9 (72.6-789.6) | 181±95.5 |  | 34.5 (4.8-1801.0) | 141.4±275.3 |
| Clear cell carcinoma (CCC) | 243.4 (80.9-5573.0) | 508.2±812.0 |  | 51.6 (3.9-9566.0) | 316.4±1167.0 |
| Non-CCC epithelial ovarian carcinoma (EOC) | 206.3 (78.3-3086.0) | 265.4±289.1 |  | 206.3 (78.3-3086.0) | 1621.0±3652.0 |
| Non-CCC EOC+BOT | 185.6 (72.6-3086.0) | 238.6±247.7 |  | 209.8 (4.8-25030.0) | 1152.0±3096.0 |
| Subgroups |  |  |  |  |  |
| Endometriosis | 144.3 (76.7-230.6) | 145.5 ± 33.7 |  | 56.2 (7.4-335.4) | 85.5±84.8 |
| Non-EMS benign lesion | 150.1 (68.3-348.8) | 158.1±50.3 |  | 14.3 (4.3-3246.0) | 92.5±433.2 |
| Stage I CCC | 207.0 (80.9-5573) | 418.4±870.8 |  | 35.0 (3.9- 378.0) | 135.2±271.7 |
| Stage II–IV CCC | 499.9 (152.6-2204) | 713.5±629.3 |  | 259.3 (18.8-9566.0) | 730.6±2048.0 |
| Stage I serous carcinoma | 166.5 (115.4-271.0) | 182.3±50.7 |  | 258.6 (6.5-1579.0) | 471.3±562.5 |
| Stage II–IV serous carcinoma | 240.4 (106.2-1189.0) | 297.3±211.5 |  | 1415 (20.4-25030.0) | 2884±5063 |
| Endometrial carcinoma | 166.8 (78.3-3086.0) | 263.2±526.4 |  | 286.9 (7.0-9477.0) | 974.8±1930.0 |
| Mucinous carcinoma | 181.3 (123.2-565.8) | 208.2±92.6 |  | 100.8 (11.9-534.7) | 173.0±173.8 |
| Other EOC | 232.3 (139.6-595.6) | 271.3±124.1 |  | 198.5 (6.6-6504.0) | 898.5±1708.0 |
| TFPI2, tissue factor pathway inhibitor 2; CA125, serum cancer antigen 125; BOT, borderline ovarian tumor; CCC, clear cell carcinoma; EOC, epithelial ovarian carcinoma; EMS, endometriosis. | | | | | |

| **Supplementary Table 3** Comparison of performance of tissue factor pathway inhibitor 2 and CA125 in discriminating Stage I and II–IV CCC patients from patients with non-clear cell epithelial ovarian carcinoma and borderline ovarian tumor | | | | |
| --- | --- | --- | --- | --- |
| **Discrimination (No. of samples)** | **Serum marker (Cutoff value)** | **AUC (95% CI)** | **Sensitivity % (95% CI)** | **Specificity % (95% CI)** |
| CCC Stage I (48) or II–IV (21)  vs BOT + non-CCC EOC (205) | TFPI2 (270 pg/mL) | Stage I: 0.581 (0.486–0.676) | 33.3 (20.4–48.4) | 79.5 (73.3–84.8) |
|  |  | Stage II–IV: 0.815 (0.711–0.920) | 66.7 (43.0–85.4) | 79.5 (73.3–84.8) |
|  | CA125 (35 U/mL) | Stage I: 0.723 (0.650–0.796) | 50.0 ( 35.2–64.8) | 24.9 (19.1–31.4) |
|  |  | Stage II–IV: 0.505 (0.401-0.610) | 90.5 (69.6–98.8) | 24.9 (19.1–31.4) |
| TFPI2, tissue factor pathway inhibitor 2; CA125, serum cancer antigen 125; BOT, borderline ovarian tumor; CCC, clear cell carcinoma; EOC, epithelial ovarian carcinoma; AUC, area under the curve. | | | | |

| **Supplementary Table 4** Comparison of performances of tissue factor pathway inhibitor 2 and CA125 in discriminating clear cell carcinoma from ovarian endometriosis | | | | |
| --- | --- | --- | --- | --- |
| **Discrimination (No. of samples)** | **Serum marker (Cutoff value)** | **AUC (95% CI)** | **Sensitivity % (95% CI)** | **Specificity % (95% CI)** |
| CCC Stage I (48) or II–IV (21)  vs EMS (21) | TFPI2 (270 pg/mL) | Stage I: 0.811 (0.710–0.911) | 33.3 (20.4–48.4) | 100 (83.9–100) |
|  |  | Stage II–IV: 0.957 (0.905–1.00) | 66.7 (43.0–85.4) | 100 (83.9–100) |
|  | CA125 (35 U/mL) | Stage I: 0.579 (0.436–0.723) | 50.0 ( 35.2–64.8) | 28.6 (11.3–52.2) |
|  |  | Stage II–IV: 0.748 (0.598–0.899) | 90.5 (69.6–98.8) | 28.6 (11.3–52.2) |
| TFPI2, tissue factor pathway inhibitor 2; CA125, serum cancer antigen 125; CCC, clear cell carcinoma; EMS, endometriosis; AUC, area under the curve. | | | | |
